# A new "American" subgroup of African-lineage Chikungunya virus detected in and isolated from mosquitoes collected in Haiti, 2016

**DOI:** 10.1371/journal.pone.0196857

**Published:** 2018-05-10

**Authors:** Sarah Keller White, Carla Mavian, Marco Salemi, John Glenn Morris, Maha A. Elbadry, Bernard A. Okech, John A. Lednicky, James C. Dunford

**Affiliations:** 1 Emerging Pathogens Institute, University of Florida, Gainesville, Florida, United States of America; 2 Department of Environmental and Global Health, College of Public Health and Health Professions, University of Florida, Gainesville, Florida, United States of America; 3 Department of Pathology, College of Medicine, University of Florida, Gainesville, Florida, United States of America; 4 Department of Medicine, College of Medicine, University of Florida, Gainesville, Florida, United States of America; 5 US Navy and Marine Corps Public Health Center, Portsmouth, Virginia, United States of America; Fundaçao Oswaldo Cruz, BRAZIL

## Abstract

As part of on-going arboviral surveillance activity in a semi-rural region in Haiti, *Chikungunya virus* (CHIKV)-positive mosquito pools were identified in 2014 (the peak of the Caribbean Asian-clade epidemic), and again in 2016 by RT-PCR. In 2014, CHIKV was only identified in *Aedes aegypti* (11 positive pools/124 screened). In contrast, in sampling in 2016, CHIKV was not identified in *Ae*. *aegypti*, but, rather, in (a) a female *Aedes albopictus* pool, and (b) a female *Culex quinquefasciatus* pool. Genomic sequence analyses indicated that the CHIKV viruses in the 2016 mosquito pools were from the East-Central-South African (ECSA) lineage, rather than the Asian lineage. In phylogenetic studies, these ECSA lineage strains form a new ECSA subgroup (subgroup IIa) together with Brazilian ECSA lineage strains from an isolated human outbreak in 2014, and a mosquito pool in 2016. Additional analyses date the most recent common ancestor of the ECSA IIa subgroup around May 2007, and the 2016 Haitian CHIKV genomes around December 2015. Known CHIKV mutations associated with improved *Ae*. *albopictus* vector competence were not identified. Isolation of this newly identified lineage from *Ae*. *albopictus* is of concern, as this vector has a broader geographic range than *Ae*. *aegypti*, especially in temperate areas of North America, and stresses the importance for continued vector surveillance.

## Introduction

From May through July 2014, a severe outbreak of Chikungunya fever (CF) occurred in Haiti, with almost 65,000 suspected cases reported to the Pan American Health Organization (PAHO) [1]. During this time, our group began a surveillance study in the Gressier region of Haiti within a population of schoolchildren diagnosed with undifferentiated febrile illness [2,3]. In this cohort, *Chikungunya virus* (CHIKV) was detected in 90 plasma specimens between May 29 and August 13, 2014. As previously reported, we obtained the complete genomic sequences of 10 CHIKV isolates from these human cases; their viral genomes belonged to the Asian lineage, and remained essentially unchanged during the three-month outbreak [4]. Only rare CF cases have occurred in our study cohort after the 2014 outbreak and the National Public Health Laboratory in Port-au-Prince has reported only two suspected CF cases to PAHO in the intervening time period [5], consistent with cessation of the initial human epidemic.

While our group, and others, have identified the CHIKV strain responsible for the 2014 Caribbean and South American epidemic as being in the Asian clade, there have been isolated reports of identification of the East-Central-South African (ECSA) lineage in Brazil, including identification in association with a localized outbreak in 2014 [6,7], and from patients and a mosquito pool in 2016 [8–10]. The Asian lineage appears to have emerged originally from the ECSA lineage, but quickly adapted to transmission in urban settings, unlike the ECSA lineage that is maintained in a sylvatic cycle and spills-over into the human population causing small localized outbreaks [11,12]. Considering that *Ae*. *aegypti* primiarly feed on humans, and are commonly found in urban settings, it is not surprising that *Ae*. *aegypti* is a successful vector of both the Asian and ECSA CHIKV lineages, whereas *Ae*. *alpopictus*, a forest dweller, is more successful in transmitting the ECSA lineage (11). Adaptive mutations of Asian lineage CHIKV (in the *envelope protein* gene segments 1 and 2, E1 and E2, including E1 T98A and A226V, and E2 L210Q) have also been identified that result in greater infectivity to mosquitoes (*Ae*. *aegypti* and *Ae*. *albopictus*) and increased vector competency of *Ae*. *albopictus* [13,14]. Another mutation, of the opal stop codon at the end of the nsP3 gene, is associated with reduction of arthralgia signs in an animal model, effecting virus pathology [15].

We report here results of screening for CHIKV in mosquito pools collected in Haiti during the 2014 CHIKV epidemic, and again in 2016. Our data document the apparent recent introduction of the “American” ECSA CHIKV lineage IIa into Haiti, and its carriage by *Ae*. *albopictus*.

## Methods

In 2014, adult *Aedes* mosquitoes were collected using Bio-Gents (BG) Sentinel traps (Bioquip, Rancho Dominguez, CA) within households and courtyards in Gressier/Leogane where children suspected of CHIKV infection resided. The traps were set from 7:00am to 6:00pm for four consecutive days. This work was approved by the University of Florida (UF) and Haitian National IRBs, and residents provided informed consents. In 2016, mosquitoes were also collected using BG Sentinel traps which were set for one day per week for twelve consecutive weeks at eight static locations within a ten mile radius in the commune of Gressier, a semi-rural setting in the Ouest department of Haiti. Trap locations were selected based on environmental considerations, security of traps, and in areas with known human arbovirus-caused illnesses. During both 2014 and 2016 trapping events, trap bags were transported to our UF BSL2-plus field laboratory in Haiti where mosquitoes were frozen at -20°C, after which they were identified by species (targeting *Ae*. *aegypti*, *Ae*. *albopictus*, *or Cx*. *quinquefasciatus*) and sexed by trained technicians using morphological keys by Leopold Rueda and Walter Reed Biosystematics Unit identification guides [16,17]. Thereafter, the mosquitoes were sorted according to location, collection date, species (*Ae*. *aegypti*, *Ae*. *albopictus*, and ‘other’ with inclusion of *Cx*. *quinquefasciatus* for 2016 collections), and sex.

The mosquitoes caught in 2014 were pooled for homogenization at the UF field laboratory in Haiti. Each pool contained 1–10 mosquitoes and was tested for CHIKV by molecular methods [18]. Mosquitoes collected in 2016 were stored at -70°C and shipped on dry ice to the Lednicky BSL3 laboratory at the Emerging Pathogens Institute at UF for further processing and virus detection and isolation. As these mosquitoes were collected in an area with previous active CHIKV transmission, and due to the possibility that viruses such as *Yellow fever virus* or other BSL3 agents may have been present in the mosquitoes, homogenization and RNA extraction at UF were performed in our BSL3 laboratory.

Mosquitoes from the 2016 collections were homogenized in refrigerated phosphate buffered saline (PBS) with two sizes of very high-density zirconium oxide beads (2mm and 0.1mm, Glen Mills, Clifton, NJ, USA) [19]. The homogenates were centrifuged, and the resulting supernatant halved: (a) one aliquot was placed in lysis buffer to initiate extraction of viral RNA (vRNA) using a Qiagen QIAamp viral RNA mini kit (Qiagen, Germantown, MD, USA), and (b) the remaining supernatant placed in PBS containing trehalose (15% (w/v) final trehalose concentration upon mixing with supernatant) for storage at -80°C for cryopreservation of virus particles for future isolation attempts in cell cultures. Each pool contained no more than 25 mosquitoes of the same species and sex, from at the same trap location. Extracted nucleic acids were subsequently screened by real-time (rt) RT-PCR for CHIKV, DENV, and ZIKV vRNAs using published protocols [18,20,21].

For the samples collected in 2016, pools that yielded a positive result for CHIKV vRNA were inoculated onto subconfluent (40%) Vero E6 cells in a 75cm^2^ flask with reduced-serum media and incubated at 37°C in 5% CO_2_ for up to 30 days for virus isolation attempts. The inoculated Vero E6 cells were refed every three days. Upon observation of virus-specific cytopathic effects (CPE) throughout 50% of the monolayer, spent media and scraped cells in spent media were collected and again tested by molecular methods for CHIKV, DENV, and ZIKV vRNAs. Additionally, the mosquito species (*Ae*. *aegypti*, *Ae*. *albopictus*, and *Cx*. *quinquefasciatus*) was confirmed in virus-positive pools by published PCR protocols [22–25]. Mosquito pools of ‘other’ species were not assessed. The homogenate and/or the spent media were used for whole genome sequencing by Sanger sequencing methods as previously reported [26] to obtain complete CHIKV genome sequences.

Pan-genomic alignment comprising of all CHIKV genomes publicly available in GenBank and the two 2016 genomes sequenced in this study were obtained using the MUSCLE algorithm implemented in MEGA7 (http://www.megasoftware.net/) [27–29]. Evidence of recombination was assessed using the set of algorithms implemented in the RDP4 software (http://web.cbio.uct.ac.za/~darren/rdp.html) [30]. Recombinant genomes were excluded from subsequent analyses. Presence of nucleotide substitution saturation was assessed using DAMBE6 (http://dambe.bio.uottawa.ca/DAMBE/) [31] and phylogenetic signal was evaluated using Tree-Puzzle (http://www.tree-puzzle.de/) [32].

Maximum likelihood (ML) phylogenetic inference was performed using the software IQ-TREE package and was based on the best-fit model chosen according to Bayesian Information Criterion [33,34]. UFBoot—Ultrafast Bootstrap (BB) Approximation (2,000 replicates) was chosen to assess statistical robustness for internal branching order in the phylogeny, and strong statistical support along the branches was defined as BB>90% [35].

The presence of temporal signal was assessed using TempEst v1.5 (http://tree.bio.ed.ac.uk/software/tempest) [36]. Time-scaled tree phylogenies were obtained performing Bayesian coalescent inference using BEAST v1.8.4 software package (http://beast.bio.ed.ac.uk), [37,38] testing the constant size demographic model against Bayesian Skyline Plot, [39] and assessing the fit of the strict or uncorrelated lognormal relaxed molecular clock model. Markov chain Monte Carlo samplers were run for 500 million generations and runs with ESS >200 (after 10% burn-in) were considered of proper mixing. The HKY substitution model [40] was used with empirical base frequencies and gamma distribution of site-specific rate of heterogeneity. Best model to fit the data was estimated by marginal likelihood estimates (MLE) obtained using path sampling and stepping-stone sampling methods [37,41]. The strength of evidence against the null hypothesis (H_0_) was evaluated via MLE comparison with the more complex model (H_A_), referred to as they Bayes Factor (BF), wherein *ln*BF<2 indicates no evidence against H_0_.

## Results

Between May and November 2014, a total of 350 mosquitoes were caught within and around 61 households in the Gressier/Leogane area, and between May and August 2016, 1756 mosquitoes were captured from eight locations in Gressier, Haiti. In rtRT-PCR screens on the year 2014 samples for CHIKV vRNA, 11 (8.9%) of 125 *Ae*. *aegypti* pools were positive, and none of 24 *Ae*. *albopictus* pools (p = 0.2. Fishers exact test, two tail). For the year 2016 samples, CHIKV vRNA was identified in 2 (1%) of 171 mosquito pools tested: (a) two female *Ae*. *albopictus* mosquitoes caught on May 17, 2016, and (b) twenty-three female *Cx*. *quinquefasciatus* caught on June 27, 2016. No CHIKV were identified in any of the 82 *Ae*. *aegypti* pools (n = 805 mosquitoes).

Upon culturing the two CHIKV-positive pools from 2016, CHIKV-induced CPE were observed 14 days post-infection of Vero cells inoculated with aliquots of the *Ae*. *albopictus* and *Cx*. *quinquefasciatus* homogenates, but not in non-inoculated controls maintained in parallel. The supernatant from both tested positive for CHIKV vRNA by rtRT-PCR, and vRNA purified from each were subsequently used for sequencing, in addition to the vRNA purified directly from mosquito homogenate from the May 17 pool (Table 1). As the mosquitoes had been identified to species by manual inspection, confirmatory testing of mosquito species in the pooled samples was accomplished using the PCR methods devised by Das *et al* [22] and by Smith *et al* [23]. The PCR tests indicated that only *Ae*. *albopictus* were present in the May 17 pool and only *Cx*. *quinquefasciatus* in the June 27 pool.

**Table 1:** Characteristics of CHIKV-positive mosquito pools, Haiti, 2016.

Pool ID	Trap location	Date collected	Mosquito sex	Mosquito species	GenBank accession number
16-5-1701	5	May 17	Female	*Aedes albopictus*	MG000876
16-5-1931	4	June 27	Female	*Culex quinquefasciatus*	MG000875

Sequencing analyses revealed that the two isolates did not contain any of the expected mutations in the E1 and E2 regions that contribute to changes in vector competency, nor changes to the opal stop codon. These sequences were highly similar to one another (99%); however, compared to previous CHIKV isolates from Haiti in 2014, the sequences were different, sharing only 93% identity.

**Fig 1:**
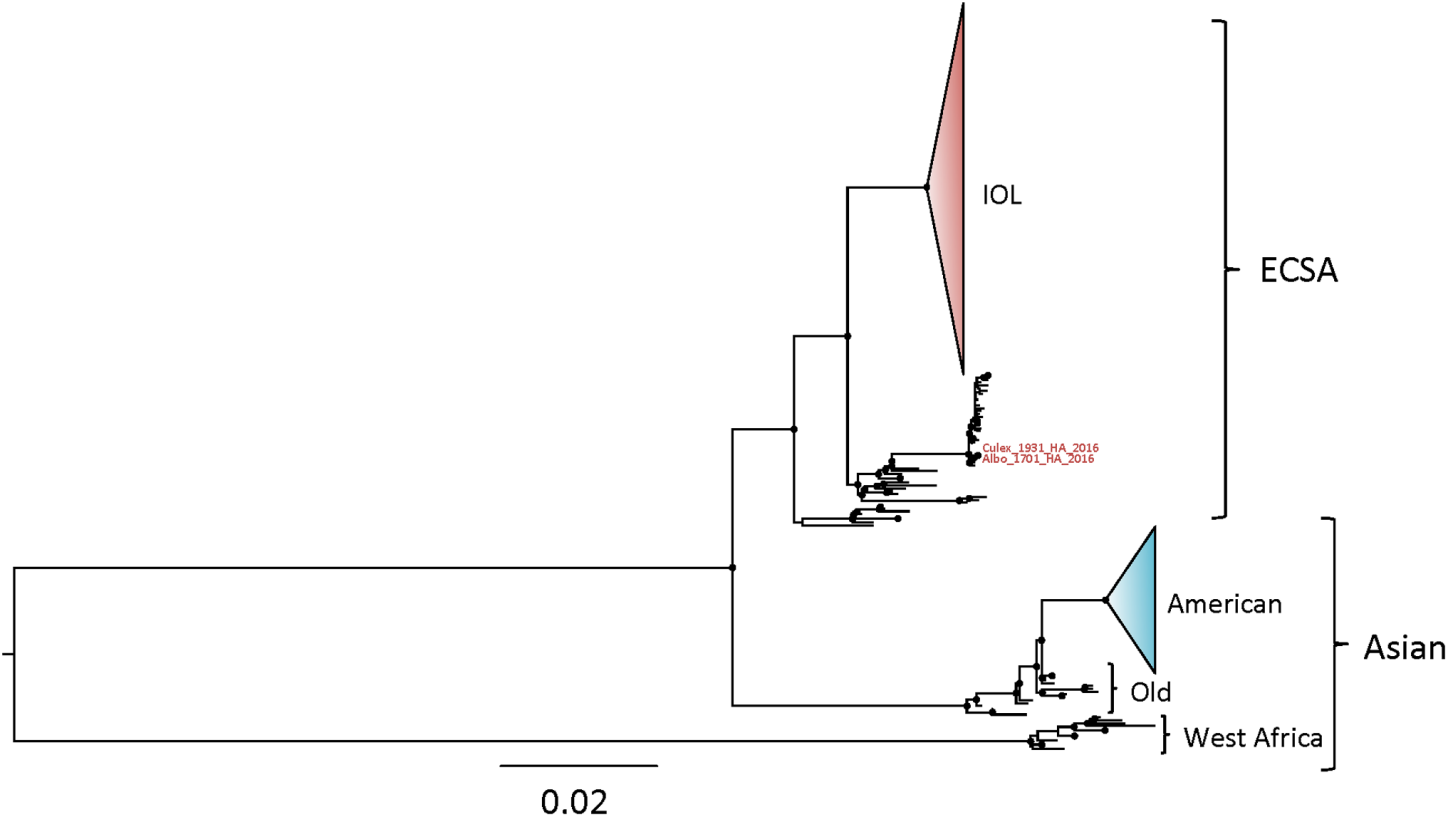
Pangenomic maximum likelihood phylogenetic inference of CHIKV. Phylogeny was inferred based on maximum likelihood method using the software IQ-TREE for the complete dataset of publicly available CHIKV genomes. Indian Ocean lineage (IOL), East/Central/South African (ECSA) lineage and Asian lineage are indicated. Branch lengths reflect genetic distances, and diamonds at each node shows strong statistical support based on ultrafast-bootstrap (BB>90%).

All sequenced CHIKV strains cluster into three main lineages: West African, East-Central-South African (ECSA), and the Asian lineage [12]. Based on our tests, no recombination (data not shown) or substitution saturation (S1A Fig) were detected, and likelihood mapping displayed relatively low phylogenetic noise (9.8%) (S1B Fig), indicating that the dataset was optimal for phylogenetic analysis. Our pan-genomic ML phylogenetic analysis of all CHIKV genomes available indicated that the two novel CHIKV genomes obtained from *Ae*. *albopictus* and *Cx*. *quinquefasciatus* mosquitoes in Haiti in 2016 belong to the ECSA lineage (Fig 1).

**Fig 2:**
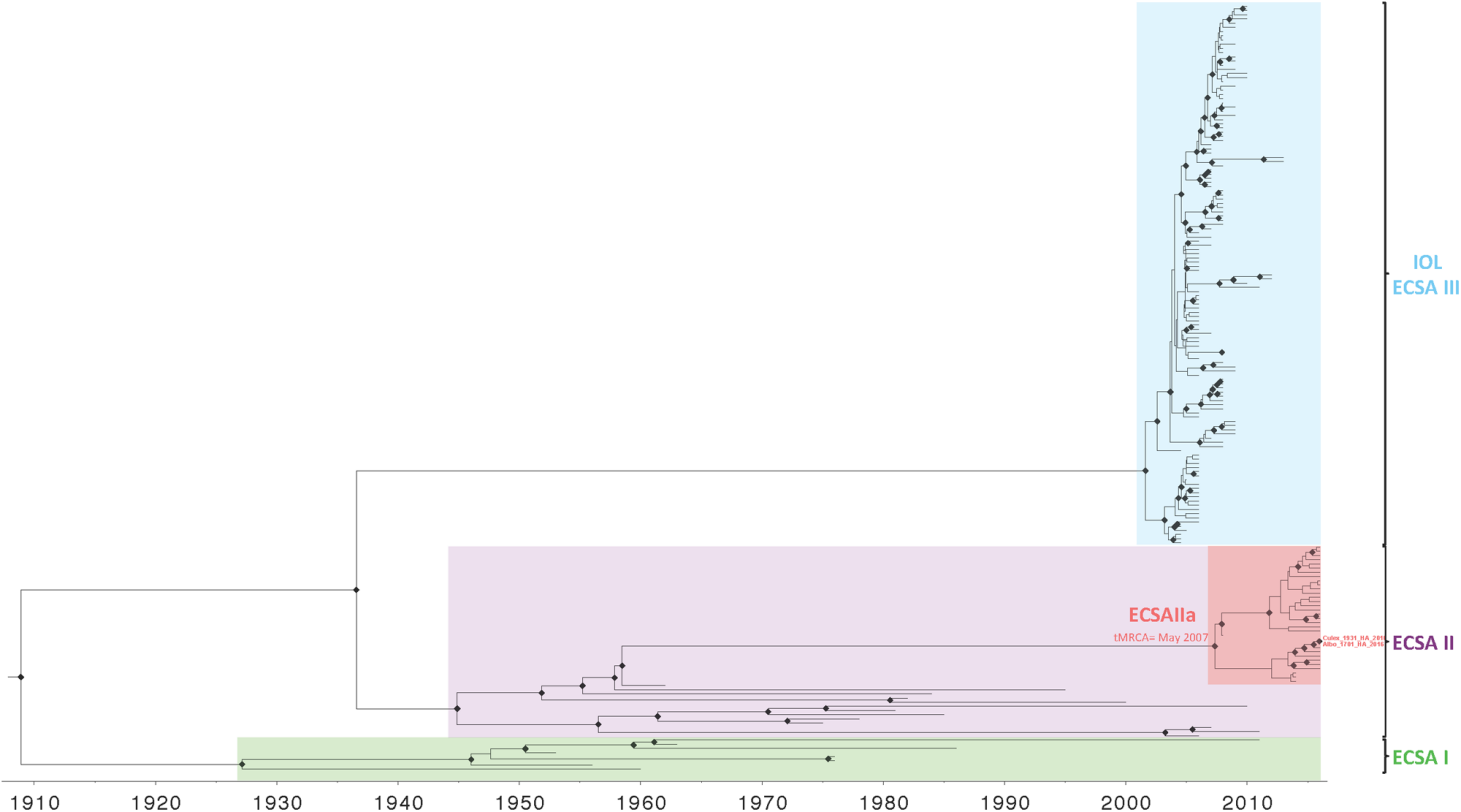
Maximum clade credibility tree of the ECSA lineages. Time-scaled phylogenetic maximum clade credibility tree inferred using the Bayesian Skyline demographic enforcing a uncorrelated lognormal relaxed clock implemented in BEAST v1.8.4. Black diamonds represent branches supported by posterior probability >0.90.

Presence of temporal signal that allows for reconstruction of the evolutionary history of the ECSA lineage was assessed (S2 Fig) before performing Bayesian coalescent phylogenetic inference. The time-scaled Maximum Clade Credibility (MCC) phylogeny of the ECSA lineage (Fig 2 and S3 Fig) was inferred using the Bayesian Skyline demographic enforcing an uncorrelated lognormal relaxed clock as determined by model testing (S1 Table). While the 2013–2014 CHIKV outbreak in the Americas aligned with the Asian lineage, our MCC phylogeny shows, in accordance with the ML phylogeny, that the novel CHIKV genomes obtained in Haiti cluster together with strains isolated in Brazil in 2014 [7] that belong to the ECSA lineage (Fig 2 and S3 Fig). The MCC tree portrayed the clear distinction between the subgroups ECSA isolates from Africa (ECSA I and II), and from the Indian Ocean region (IOL) (ECSA III) [42,43], and a new distinct ECSA subgroup IIa (ECSA IIa) arising from the ECSA II lineage (Fig 2 and S3 Fig).

The estimated time of the most recent common ancestor (tMRCA) for the new ECSA IIa subgroup was found to be May 2007 with a 95% highest posterior density (HPD) interval of April 2006 –January 2008. The MCC tree shows presence of two clades within the ECSA IIa lineage, one of which contained both Brazilian and Haitian sequences. The tMRCA for this clade was December 2013 (HPD 95% interval of April 2012 –February 2015). The Haitian strains obtained in this study share a common ancestor that was dated around December 2015 with a HPD 95% interval of October 2015 –January 2016, suggesting recent introduction to the country.

## Discussion

Here we report the first detection of the CHIKV ECSA lineage in Haiti, with our Haitian strains forming a new ECSA subgroup IIa together with CHIKV strains previously reported from Brazil [8,9]. Our molecular clock analysis suggests that this “American” ECSA lineage diverged from the African ECSA lineages sometime in the range of mid-2012-early 2015, within the range of the time period when the major Asian-clade CHIKV epidemic started in the Americas. Our analyses further suggest that the Haiti ECSA lineage IIa strain diverged from the earlier Brazilian strains sometime between October, 2015 and January, 2016, suggesting that it was introduced into Haiti after passage of the main CHIKV Asian clade epidemic. We have previously noted what appears to have been transfer of arbovirus strains between Brazil and Haiti [44,45]; under these circumstances, movement of the ECSA CHIKV strain from Brazil to Haiti in the time period noted would clearly be plausible.

Not unexpectedly, given the massive size of the initial CHIKV epidemic in Haiti and the Caribbean, our 2014 studies documented CHIKV in close to 9% of the *Ae*. *aegypti* pools sampled. In contrast, we were not able to identify CHIKV in any of the 24 *Ae*. *albopictus* pools collected in 2014. While numbers are small and differences between rates of *Ae*. *aegypti* and *Ae*. *albopictus* identification are not statistically significant, these findings lend credence to the idea that *Ae*. *aegypti* was the primary vector for the Asian clade epidemic strain. In contrast, in 2016, the two CHIKV ECSA lineage IIa strains identified were from *Ae*. *albopictus* and *Culex quinquefasciatus*–with no identification in any *Ae*. *aegypti* pools. Again, numbers are small; however, our findings raise the possibility that *Ae*. *albopictus*, even in the absence of genetic changes that have been associated with increased *Ae*. *albopictus* transmission, plays a more important role in transmission of this new clade than does *Ae*. *aegypti*. This is of potential public health concern, given that *Ae*. *albopictus* is highly prevalent in the Caribbean and the Americas, with a range that reaches further into temperate regions of the United States than is seen with *Ae*. *aegypti* [11,46]. While *Cx*. *quinquefasciatus* was defined as a poor CHIKV vector in one study [47], this concept should also be re-examined in contemporary terms with the viruses in circulation and relevant mosquito subspecies. It is plausible that the CHIKV genome could adapt for enhanced vector to human transmission by *Cx*. *quinquefasciatus*, particularly as others have detected CHIKV in wild-caught *Cx*. *quinquefasciatus* [48].

There are some limitations when conducting mosquito surveillance efforts and utilizing wild-caught mosquitoes for virus detection. It is possible that during tests of our mosquito pools additional CHIKV-positive pools were missed due to the limits of detection by rtRT-PCR. We have found on numerous occasions that virus isolation in cell cultures enhances the ability to identify virus-positive samples when the viral loads are too low for rtRT-PCR, but virus isolation is resource-intensive and impractical for every mosquito pool. To further improve our chances of virus detection, excess PBS is not used during our mosquito homogenization protocol so as not to dilute the concentration of virus in the homogenates, as that negatively impacts downstream applications such as detection by RT-PCR and virus isolation in cultured cells. CHIKV was detected in approximately 1% of all wild-caught mosquito pools identified by species and tested for CHIKV vRNA (2/171 x 100) in the 2016 portion of the study. This detection rate is ten-fold greater than a previous estimate for the natural infection rate for CHIKV in *Ae*. *aegypti* and *Ae*. *albopictus* mosquitoes [49], underscoring the potential utility of our approach.

Given the high levels of infection seen with the 2014 CHIKV Asian clade epidemic in the Caribbean and South America, it is unlikely that we will see another major Asian clade epidemic in the near future. However, we are seeing a very different pattern with the CHIKV ECSA lineage IIa strains, with only small numbers of reported cases and localized outbreaks [9,10]. This would be consistent with some level of endemicity in either the vector population or a natural reservoir, possibly within a sylvatic cycle similar to what has been reported in Africa. While there are no nonhuman primates to serve as a CHIKV reservoir in Haiti, other mammals and some birds have been noted as potential reservoirs [50]. Of additional concern, studies of wild-caught mosquitoes have generated evidence of vertical transmission in both *Ae*. *aegypti* and *Ae*. *albopictus* [51–53], which indicates that CHIKV can be maintained within the mosquito population until human immunity wanes over time and another outbreak can occur. This, in turn, underscores the importance of continuing vector surveillance and screening for clinical CHIKV infections, to detect possible ongoing endemic infection, outside of epidemic settings.

## Supporting information

S1 Fig Substitution saturation and phylogenetic signal for pan-genomic CHIKV dataset. (A) Scatter plot of nucleotide transition (s) and transversion (v) substitutions over genetic distance measured by TN93 nucleotide substitution model. (B) Likelihood triangle showing supports for each of three alternative topologies (tips), unresolved quartets (center) and partly resolved quartets (edges).
(TIF)

S2 Fig Assessment of temporal signal. The plot represents regression analysis of root-to-tip genetic distance for the ECSA lineage assessed using TempEst v1.5. The positive slope and the correlation coefficient “r” indicate presence of temporal signal for the dataset.
(TIF)

S3 Fig ECSA maximum clade credibility tree of the ECSA lineages with tips. Time-scaled phylogenetic maximum clade credibility tree inferred using the Bayesian Skyline demographic enforcing a uncorrelated lognormal relaxed clock implemented in BEAST v1.8.4. Black diamonds represent branches supported by posterior probability >0.90.
(TIFF)

S1 Table Molecular clock and demographic tree prior model comparison. (DOCX)

## References

[1] Organization PAH (2015) Number of reported cases of chikungunya fever in the Americas, by country or territory, 2013–2014. Geneva, Switzerland.

[2] Beau de Rochars VM, Elbadry M, Ball J, Telisma T, Chavannes S, et al. (2017) Clinical findings among laboratory-confirmed cases of Chikungunya infection in a naive student cohort in Haiti. Submitted for publication.

[3] Beau De Rochars VE, Alam MT, Telisma T, Masse R, Chavannes S, et al (2015) Spectrum of outpatient illness in a school-based cohort in Haiti, with a focus on diarrheal pathogens. Am J Trop Med Hyg 92: 752–757. doi: 10.4269/ajtmh.14-0059 25732684 10.4269/ajtmh.14-0059 PMC4385768

[4] White SK, Morris JG, Elbadry MA, Beau De Rochars VM, Okech BA, et al (2017) Complete Genome Sequences of Chikungunya Viruses Isolated from Plasma Specimens Collected from Haitians in 2014. Genome Announc 5. 10.1128/genomeA.00148-17 PMC5391409 28408671

[5] Organization PAH (2016) Number of reported cases of chikungunya fever in the Americas, by country or territory, 2016 EW 27. Geneva, Switzerland.

[6] Teixeira MG, Andrade AM, Costa MaC, Castro JN, Oliveira FL, et al (2015) East/Central/South African genotype chikungunya virus, Brazil, 2014. Emerg Infect Dis 21: 906–907. doi: 10.3201/eid2105.141727 25898939 10.3201/eid2105.141727 PMC4412231

[7] Nunes MR, Faria NR, de Vasconcelos JM, Golding N, Kraemer MU, et al (2015) Emergence and potential for spread of Chikungunya virus in Brazil. BMC Med 13: 102 doi: 10.1186/s12916-015-0348-x 25976325 10.1186/s12916-015-0348-x PMC4433093

[8] Cunha MS, Cruz NVG, Schnellrath LC, Medaglia MLG, Casotto ME, et al (2017) Autochthonous Transmission of East/Central/South African Genotype Chikungunya Virus, Brazil. Emerg Infect Dis 23: 1737–1739. doi: 10.3201/eid2310.161855 28930027 10.3201/eid2310.161855 PMC5621531

[9] Charlys da Costa A, Theze J, Komninakis SCV, Sanz-Duro RL, Felinto MRL, et al (2017) Spread of Chikungunya Virus East/Central/South African Genotype in Northeast Brazil. Emerg Infect Dis 23: 1742–1744. doi: 10.3201/eid2310.170307 28930031 10.3201/eid2310.170307 PMC5621546

[10] Costa-da-Silva AL, Ioshino RS, Petersen V, Lima AF, Cunha MDP, et al (2017) First report of naturally infected Aedes aegypti with chikungunya virus genotype ECSA in the Americas. PLoS Negl Trop Dis 11: e0005630 doi: 10.1371/journal.pntd.0005630 28614394 10.1371/journal.pntd.0005630 PMC5470658

[11] Tsetsarkin KA, Chen R, Leal G, Forrester N, Higgs S, et al (2011) Chikungunya virus emergence is constrained in Asia by lineage-specific adaptive landscapes. Proc Natl Acad Sci U S A 108: 7872–7877. doi: 10.1073/pnas.1018344108 21518887 10.1073/pnas.1018344108 PMC3093459

[12] Powers AM, Brault AC, Tesh RB, Weaver SC (2000) Re-emergence of Chikungunya and O’nyong-nyong viruses: evidence for distinct geographical lineages and distant evolutionary relationships. J Gen Virol 81: 471–479. doi: 10.1099/0022-1317-81-2-471 10644846 10.1099/0022-1317-81-2-471

[13] Stapleford KA, Moratorio G, Henningsson R, Chen R, Matheus S, et al (2016) Whole-Genome Sequencing Analysis from the Chikungunya Virus Caribbean Outbreak Reveals Novel Evolutionary Genomic Elements. PLoS Negl Trop Dis 10: e0004402 doi: 10.1371/journal.pntd.0004402 26807575 10.1371/journal.pntd.0004402 PMC4726740

[14] Tsetsarkin KA, Weaver SC (2011) Sequential adaptive mutations enhance efficient vector switching by Chikungunya virus and its epidemic emergence. PLoS Pathog 7: e1002412 doi: 10.1371/journal.ppat.1002412 22174678 10.1371/journal.ppat.1002412 PMC3234230

[15] Jones JE, Long KM, Whitmore AC, Sanders W, Thurlow LR, et al (2017) Diruption of the opal stop codon attenuates Chikungunya virus-induced arthritis and pathology. mBio 8: e01456–01417. doi: 10.1128/mBio.01456-17 29138302 10.1128/mBio.01456-17 PMC5686535

[16] Unit WRB Mosquito ID: Culex (Cux.) quinquefasciatus. Silver Spring, MD: The Walter Reed Biosystematics Unit.

[17] Rueda LM (2004) Pictoral keys for the identification of mosquitoes (Diptera: Culicidae) associated with Dengue Virus Transmission. pp. 33–41.

[18] Lanciotti RS, Kosoy OL, Laven JJ, Panella AJ, Velez JO, et al (2007) Chikungunya virus in US travelers returning from India, 2006. Emerg Infect Dis 13: 764–767. doi: 10.3201/eid1305.070015 17553261 10.3201/eid1305.070015 PMC2738459

[19] Crowder CD, Rounds MA, Phillipson CA, Picuri JM, Matthews HE, et al (2010) Extraction of total nucleic acids from ticks for the detection of bacterial and viral pathogens. J Med Entomol 47: 89–94. 20180313 10.1603/033.047.0112 PMC2837073

[20] Lanciotti RS, Kosoy OL, Laven JJ, Velez JO, Lambert AJ, et al (2008) Genetic and serologic properties of Zika virus associated with an epidemic, Yap State, Micronesia, 2007. Emerg Infect Dis 14: 1232–1239. doi: 10.3201/eid1408.080287 18680646 10.3201/eid1408.080287 PMC2600394

[21] Santiago GA, Vergne E, Quiles Y, Cosme J, Vazquez J, et al (2013) Analytical and clinical performance of the CDC real time RT-PCR assay for detection and typing of dengue virus. PLoS Negl Trop Dis 7: e2311 doi: 10.1371/journal.pntd.0002311 23875046 10.1371/journal.pntd.0002311 PMC3708876

[22] Das B, Swain S, Patra A, Das M, Tripathy HK, et al (2012) Development and evaluation of a single-step multiplex PCR to differentiate the aquatic stages of morphologically similar Aedes (subgenus: Stegomyia) species. Trop Med Int Health 17: 235–243. doi: 10.1111/j.1365-3156.2011.02899.x 22040518 10.1111/j.1365-3156.2011.02899.x

[23] Smith JL, Fonseca DM (2004) Rapid assays for identification of members of the Culex (Culex) pipiens complex, their hybrids, and other sibling species (Diptera: culicidae). Am J Trop Med Hyg 70: 339–345. 15100444

[24] Salvemini M, Mauro U, Lombardo F, Milano A, Zazzaro V, et al (2011) Genomic organization and splicing evolution of the doublesex gene, a Drosophila regulator of sexual differentiation, in the dengue and yellow fever mosquito Aedes aegypti. BMC Evol Biol 11: 41 doi: 10.1186/1471-2148-11-41 21310052 10.1186/1471-2148-11-41 PMC3045327

[25] Sirot LK, Poulson RL, McKenna MC, Girnary H, Wolfner MF, et al (2008) Identity and transfer of male reproductive gland proteins of the dengue vector mosquito, Aedes aegypti: potential tools for control of female feeding and reproduction. Insect Biochem Mol Biol 38: 176–189. doi: 10.1016/j.ibmb.2007.10.007 18207079 10.1016/j.ibmb.2007.10.007 PMC2758040

[26] Cherabuddi K, Iovine NM, Shah K, White SK, Paisie T, et al (2016) Zika and Chikungunya virus co-infection in a traveller returning from Colombia, 2016: Virus isolation and genetic analysis. JMM Case Reports: 1–6. 10.1099/jmmcr.0.005072 PMC5343122 28348794

[27] Kumar S, Stecher G, Tamura K (2016) MEGA7: Molecular Evolutionary Genetics Analysis Version 7.0 for Bigger Datasets. Mol Biol Evol 33: 1870–1874. doi: 10.1093/molbev/msw054 27004904 10.1093/molbev/msw054 PMC8210823

[28] Edgar RC (2004) MUSCLE: a multiple sequence alignment method with reduced time and space complexity. BMC Bioinformatics 5: 113 doi: 10.1186/1471-2105-5-113 15318951 10.1186/1471-2105-5-113 PMC517706

[29] Edgar RC (2004) MUSCLE: multiple sequence alignment with high accuracy and high throughput. Nucleic Acids Res 32: 1792–1797. doi: 10.1093/nar/gkh340 15034147 10.1093/nar/gkh340 PMC390337

[30] Martin DP, Murrell B, Golden M, Khoosal A, Muhire B (2015) RDP4: Detection and analysis of recombination patterns in virus genomes. Virus Evol 1: vev003 doi: 10.1093/ve/vev003 27774277 10.1093/ve/vev003 PMC5014473

[31] Xia X, Xie Z (2001) DAMBE: software package for data analysis in molecular biology and evolution. J Hered 92: 371–373. 11535656 10.1093/jhered/92.4.371

[32] Schmidt HA, Strimmer K, Vingron M, von Haeseler A (2002) TREE-PUZZLE: maximum likelihood phylogenetic analysis using quartets and parallel computing. Bioinformatics 18: 502–504. 11934758 10.1093/bioinformatics/18.3.502

[33] Nguyen LT, Schmidt HA, von Haeseler A, Minh BQ (2015) IQ-TREE: a fast and effective stochastic algorithm for estimating maximum-likelihood phylogenies. Mol Biol Evol 32: 268–274. doi: 10.1093/molbev/msu300 25371430 10.1093/molbev/msu300 PMC4271533

[34] Trifinopoulos J, Nguyen LT, von Haeseler A, Minh BQ (2016) W-IQ-TREE: a fast online phylogenetic tool for maximum likelihood analysis. Nucleic Acids Res 44: W232–235. doi: 10.1093/nar/gkw256 27084950 10.1093/nar/gkw256 PMC4987875

[35] Minh BQ, Nguyen MA, von Haeseler A (2013) Ultrafast approximation for phylogenetic bootstrap. Mol Biol Evol 30: 1188–1195. doi: 10.1093/molbev/mst024 23418397 10.1093/molbev/mst024 PMC3670741

[36] Rambaut A, Lam TT, Max Carvalho L, Pybus OG (2016) Exploring the temporal structure of heterochronous sequences using TempEst (formerly Path-O-Gen). Virus Evol 2: vew007 doi: 10.1093/ve/vew007 27774300 10.1093/ve/vew007 PMC4989882

[37] Drummond AJ, Rambaut A (2007) BEAST: Bayesian evolutionary analysis by sampling trees. BMC Evol Biol 7: 214 doi: 10.1186/1471-2148-7-214 17996036 10.1186/1471-2148-7-214 PMC2247476

[38] Drummond AJ, Suchard MA, Xie D, Rambaut A (2012) Bayesian phylogenetics with BEAUti and the BEAST 1.7. Mol Biol Evol 29: 1969–1973. doi: 10.1093/molbev/mss075 22367748 10.1093/molbev/mss075 PMC3408070

[39] Strimmer K, Pybus OG (2001) Exploring the demographic history of DNA sequences using the generalized skyline plot. Mol Biol Evol 18: 2298–2305. doi: 10.1093/oxfordjournals.molbev.a003776 11719579 10.1093/oxfordjournals.molbev.a003776

[40] Hasegawa M, Kishino H, Yano T (1985) Dating of the human-ape splitting by a molecular clock of mitochondrial DNA. J Mol Evol 22: 160–174. 3934395 10.1007/BF02101694

[41] Baele G, Lemey P, Bedford T, Rambaut A, Suchard MA, et al (2012) Improving the accuracy of demographic and molecular clock model comparison while accommodating phylogenetic uncertainty. Mol Biol Evol 29: 2157–2167. doi: 10.1093/molbev/mss084 22403239 10.1093/molbev/mss084 PMC3424409

[42] Nasci RS (2014) Movement of chikungunya virus into the Western hemisphere. Emerg Infect Dis 20: 1394–1395. doi: 10.3201/eid2008.140333 25061832 10.3201/eid2008.140333 PMC4111178

[43] Arankalle VA, Shrivastava S, Cherian S, Gunjikar RS, Walimbe AM, et al (2007) Genetic divergence of Chikungunya viruses in India (1963–2006) with special reference to the 2005–2006 explosive epidemic. J Gen Virol 88: 1967–1976. doi: 10.1099/vir.0.82714-0 17554030 10.1099/vir.0.82714-0

[44] Lednicky J, Beau De Rochars VM, El Badry M, Loeb J, Telisma T, et al (2016) Zika Virus Outbreak in Haiti in 2014: Molecular and Clinical Data. PLoS Negl Trop Dis 10: e0004687 doi: 10.1371/journal.pntd.0004687 27111294 10.1371/journal.pntd.0004687 PMC4844159

[45] Lednicky J, De Rochars V, Elbadry M, Loeb J, Telisma T, et al (2016) Mayaro virus in child with acute febrile illness, Haiti, 2015. Emerg Infect Dis 22: 2000–2002. doi: 10.3201/eid2211.161015 27767924 10.3201/eid2211.161015 PMC5088037

[46] Weaver SC, Lecuit M (2015) Chikungunya virus and the global spread of a mosquito-borne disease. N Engl J Med 372: 1231–1239. doi: 10.1056/NEJMra1406035 25806915 10.1056/NEJMra1406035

[47] Jupp PG, McIntosh BM, Dos Santos I, DeMoor P (1981) Laboratory vector studies on six mosquito and one tick species with chikungunya virus. Trans R Soc Trop Med Hyg 75: 15–19. 6115488 10.1016/0035-9203(81)90005-5

[48] Bessaud M, Peyrefitte CN, Pastorino BA, Tock F, Merle O, et al (2006) Chikungunya virus strains, Reunion Island outbreak. Emerg Infect Dis 12: 1604–1606. doi: 10.3201/eid1210.060596 17176585 10.3201/eid1210.060596 PMC3290959

[49] Gu W, Novak RJ (2004) Short report: detection probability of arbovirus infection in mosquito populations. Am J Trop Med Hyg 71: 636–638. 15569797

[50] Caglioti C, Lalle E, Castilletti C, Carletti F, Capobianchi MR, et al (2013) Chikungunya virus infection: an overview. New Microbiol 36: 211–227. 23912863

[51] Thavara U, Tawatsin A, Pengsakul T, Bhakdeenuan P, Chanama S, et al (2009) Outbreak of chikungunya fever in Thailand and virus detection in field population of vector mosquitoes, Aedes aegypti (L.) and Aedes albopictus Skuse (Diptera: Culicidae). Southeast Asian J Trop Med Public Health 40: 951–962. 19842379

[52] Ratsitorahina M, Harisoa J, Ratovonjato J, Biacabe S, Reynes JM, et al (2008) Outbreak of dengue and Chikungunya fevers, Toamasina, Madagascar, 2006. Emerg Infect Dis 14: 1135–1137. doi: 10.3201/eid1407.071521 18598641 10.3201/eid1407.071521 PMC2600361

[53] Delatte H, Paupy C, Dehecq JS, Thiria J, Failloux AB, et al (2008) [Aedes albopictus, vector of chikungunya and dengue viruses in Reunion Island: biology and control]. Parasite 15: 3–13. doi: 10.1051/parasite/2008151003 18416242 10.1051/parasite/2008151003

