# Supplementary figures and images for: A new "American" subgroup of African-lineage Chikungunya virus detected in and isolated from mosquitoes collected in Haiti, 2016
Source: PLoS One. 2018 May 10;13(5):e0196857. doi: 10.1371/journal.pone.0196857 (PMC5944945; doi:10.1371/journal.pone.0196857)

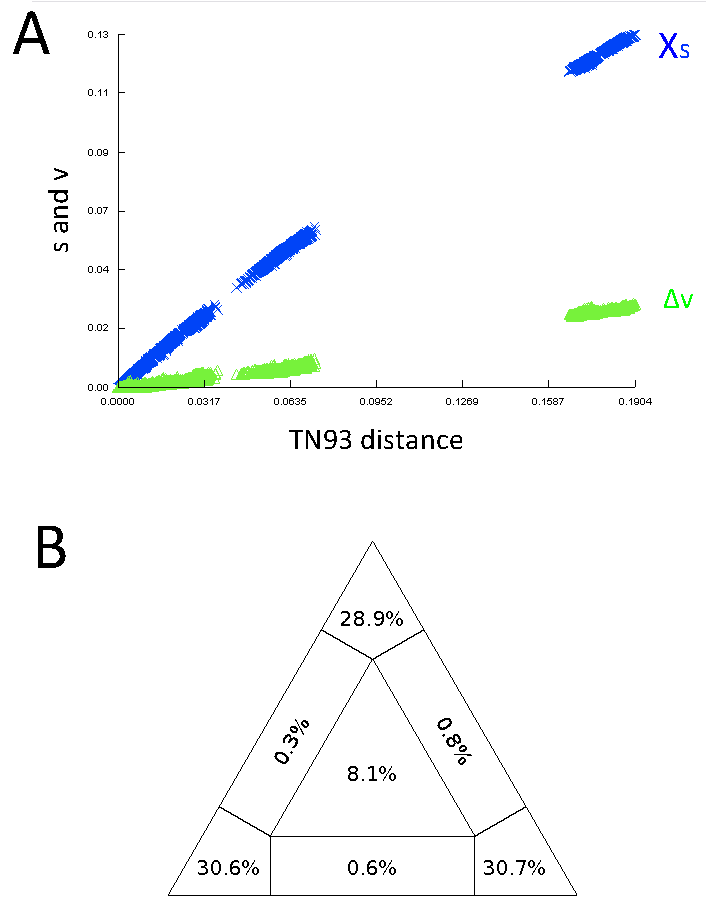

Supplement: S1 Fig — (A) Scatter plot of nucleotide transition (s) and transversion (v) substitutions over genetic distance measured by TN93 nucleotide substitution model. (B) Likelihood triangle showing supports for each of three alternative topologies (tips), unresolved quartets (center) and partly resolved quartets (edges). (TIF) [file pone.0196857.s001.tif]

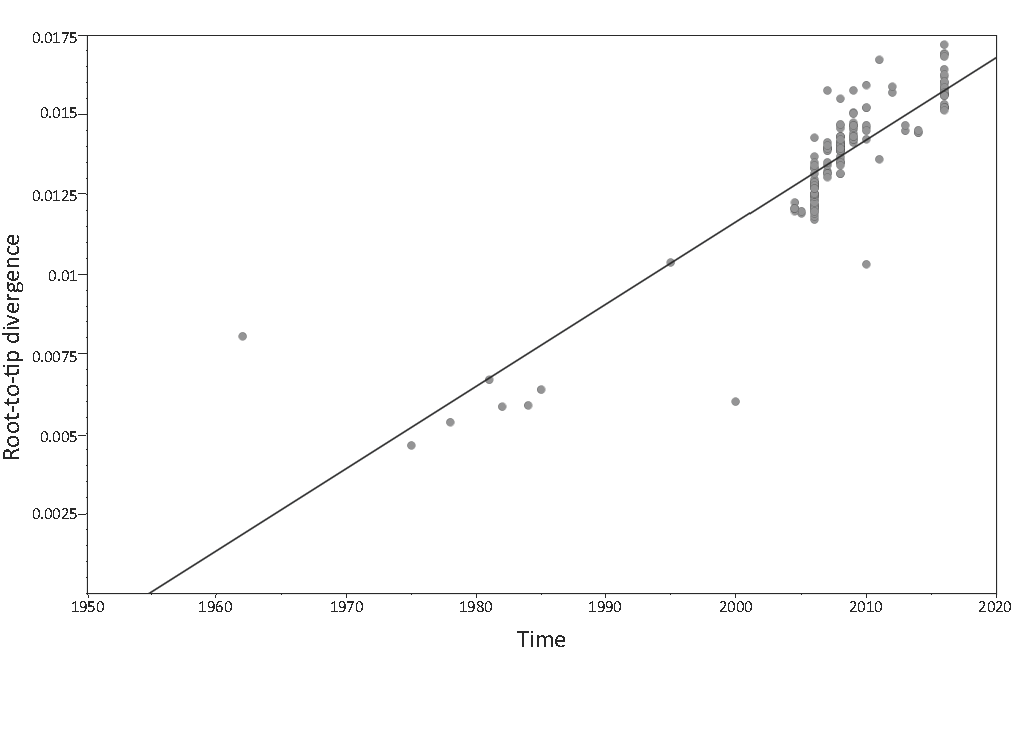

Supplement: S2 Fig — The plot represents regression analysis of root-to-tip genetic distance for the ECSA lineage assessed using TempEst v1.5. The positive slope and the correlation coefficient “r” indicate presence of temporal signal for the dataset. (TIF) [file pone.0196857.s002.tif]

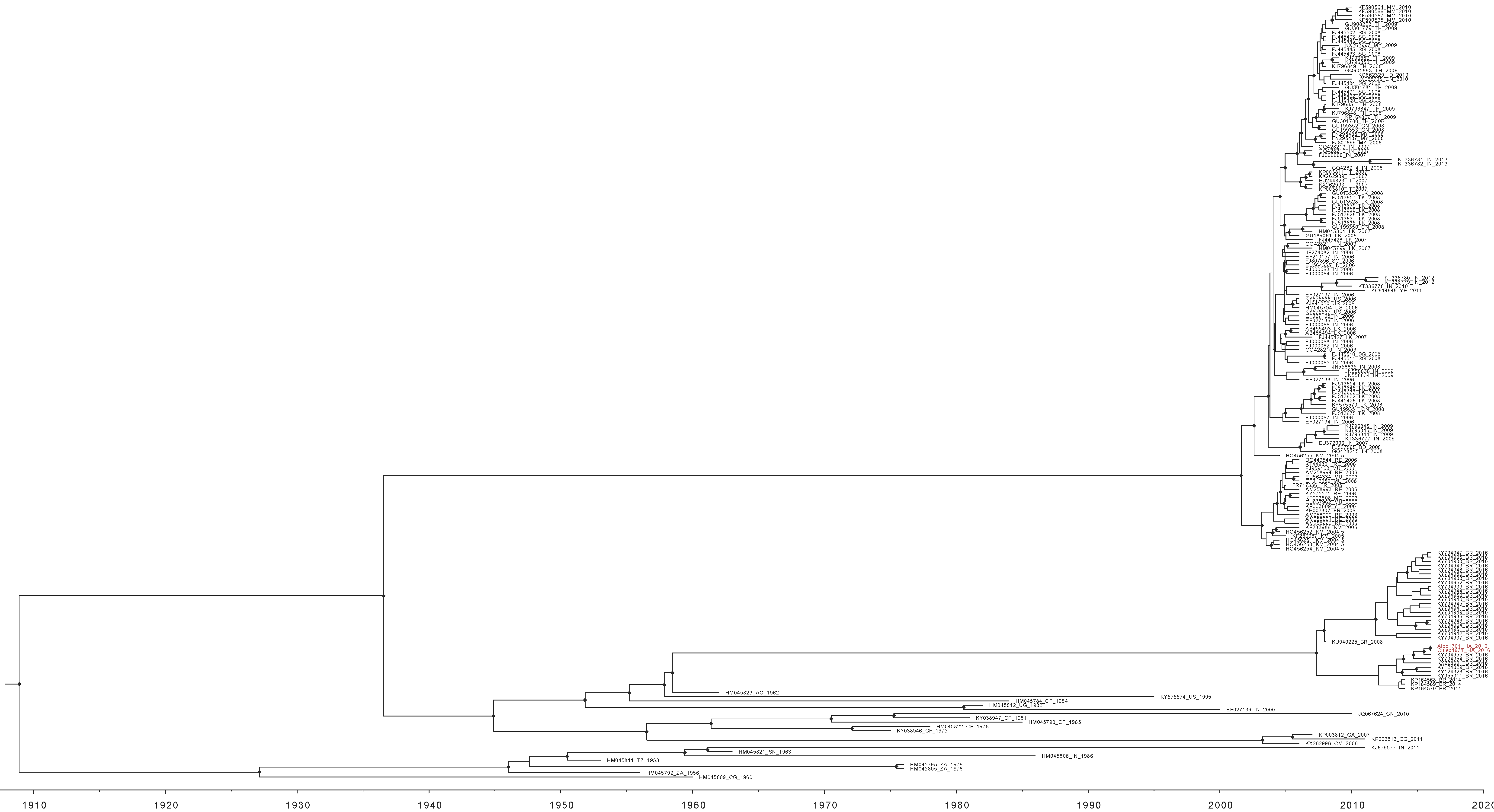

Supplement: S3 Fig — Time-scaled phylogenetic maximum clade credibility tree inferred using the Bayesian Skyline demographic enforcing a uncorrelated lognormal relaxed clock implemented in BEAST v1.8.4. Black diamonds represent branches supported by posterior probability >0.90. (TIFF) [file pone.0196857.s003.tiff]
