# Supplementary material for: A new "American" subgroup of African-lineage Chikungunya virus detected in and isolated from mosquitoes collected in Haiti, 2016
Source: PLoS One. 2018 May 10;13(5):e0196857. doi: 10.1371/journal.pone.0196857 (PMC5944945; doi:10.1371/journal.pone.0196857)
Supplement: S1 Table — (DOCX) [file pone.0196857.s004.docx]

**Table S1. Molecular clock and demographic tree prior model comparison**

| **MODEL** | **SS** | **Ln(BF)ss** | **PS** | **Ln(BF)ps** |
| --- | --- | --- | --- | --- |
| SC Const | -31080.01837 |  | -31079.56981 |  |
| RC Const | -30945.57501 | 134 | -30945.45909 | 134 |
| RC Const | -30945.57501 |  | -30945.45909 |  |
| **RC Bayesian Skyline Plot** | -30933.43444 | 12 | -30931.93791 | 13 |

SS=stepping stone, PS= path sampling. The best model fitting the data is highlighted in bold.
